# Vinculin controls talin engagement with the actomyosin machinery

**DOI:** 10.1038/ncomms10038

**Published:** 2015-12-04

**Authors:** Paul Atherton, Ben Stutchbury, De-Yao Wang, Devina Jethwa, Ricky Tsang, Eugenia Meiler-Rodriguez, Pengbo Wang, Neil Bate, Roy Zent, Igor L. Barsukov, Benjamin T. Goult, David R. Critchley, Christoph Ballestrem

**Affiliations:** 1 Wellcome Trust Centre for Cell-Matrix Research, University of Manchester, Manchester M13 9PT, UK; 2 Department of Microbiology, Complutense, University of Madrid, 28040 Madrid, Spain; 3 Department of Biochemistry, University of Leicester, Lancaster Road, Leicester LE1 9HN, UK; 4 Vanderbilt Centre for Kidney Disease, Vanderbilt Division of Nephrology, S-3223 Medical Centre, North Nashville, Tennessee, USA; 5 Institute of Integrative Biology, University of Liverpool, Crown Street, Liverpool L69 7ZB, UK; 6 School of Biosciences, University of Kent, Canterbury, KENT CT2 7NJ, UK

## Abstract

The link between extracellular-matrix-bound integrins and intracellular F-actin is essential for cell spreading and migration. Here, we demonstrate how the actin-binding proteins talin and vinculin cooperate to provide this link. By expressing structure-based talin mutants in talin null cells, we show that while the C-terminal actin-binding site (ABS3) in talin is required for adhesion complex assembly, the central ABS2 is essential for focal adhesion (FA) maturation. Thus, although ABS2 mutants support cell spreading, the cells lack FAs, fail to polarize and exert reduced force on the surrounding matrix. ABS2 is inhibited by the preceding mechanosensitive vinculin-binding R3 domain, and deletion of R2R3 or expression of constitutively active vinculin generates stable force-independent FAs, although cell polarity is compromised. Our data suggest a model whereby force acting on integrin-talin complexes via ABS3 promotes R3 unfolding and vinculin binding, activating ABS2 and locking talin into an actin-binding configuration that stabilizes FAs.

## Abstract (web summary)

The mechanosensitive proteins talin and vinculin mediate the linkage between integrin-bound extracellular matrix and the actin cytoskeleton. Here the authors dissect distinct roles for two actin-binding sites within talin on adhesion complex assembly and maturation, which are regulated by vinculin binding to talin.

## Graphical abstract

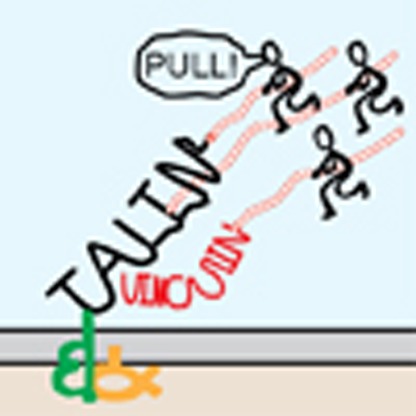

Cell motility is central to the development and homeostasis of multicellular organisms, and defining the mechanisms involved will inform strategies to modulate aberrant cell migration and promote tissue regeneration. Cell migration involves the cyclical attachment and detachment of the integrin family of adhesion molecules to extracellular matrix (ECM), as well as the generation of force required to translocate the cell body. Such events occur in focal adhesions (FA), dynamic macromolecular complexes in which integrins are linked via cytoplasmic adhesion plaque proteins to the actomyosin contractile machinery^1^,^2^,^3^. Two plaque proteins that are key to this link are the interacting actin-binding proteins talin and vinculin. Cells depleted of talin cannot maintain cell spreading^4^, while cells without vinculin have smaller more dynamic FAs^1^,^2^,^3^,^5^,^6^ and are compromised in coupling plaque proteins to F-actin^4^,^7^,^8^.

Structurally, talin consists of an atypical N-terminal FERM-domain (talin head) that binds integrins, PIP2 and F-actin (actin-binding site 1; ABS1) linked to a C-terminal flexible rod consisting of 13 alpha-helical bundles (R1-R13) terminating in a dimerization motif (Fig. 1a)^9^. The rod contains binding sites for the Rap1-interactor RIAM^10^,^11^, vinculin^12^ and integrins^13^ plus two additional regions that bind F-actin (ABS2 and ABS3)^1^,^2^,^3^,^14^. Binding of the talin head to integrins regulates their affinity for ECM^4^,^15^, while talin binding to actin is thought to form the primary link to the force-transmitting machinery^4^,^5^,^6^,^9^. Vinculin, which binds talin via its globular N-terminal head and F-actin via its C-terminal tail, acts as a molecular clutch, reinforcing the link between talin and actomyosin^7^,^8^,^10^,^16^,^17^. Moreover, vinculin binding to talin maintains integrins in an active conformation^9^,^11^,^16^, stabilizing the entire FA structure containing a large number of signalling components^10^,^11^,^16^. However, expression of constitutively active vinculin compromises cell polarity and directional cell migration^12^,^16^. Thus, co-ordinated cell migration requires that the activity of talin and vinculin are precisely controlled, but the mechanisms that regulate assembly of the talin/vinculin complex and its interaction with the cellular force machinery remain to be explored.

The activity of talin and vinculin is regulated by conformational changes at several levels. In both cases, the N-terminal head domains interact with the C-terminal regions of the proteins resulting in an autoinhibited state^7^,^8^,^9^,^18^. Despite evidence that activation involves either chemical signals (binding to activating proteins or lipids) and/or physical force^9^,^10^,^19^, the detailed mechanisms underlying activation have not been elucidated. In addition, structural and biochemical studies on talin show that the vinculin binding sites (VBSs) within the helical bundles that make up the talin rod are cryptic^12^,^14^,^20^,^21^,^22^, and single molecule experiments^9^,^23^,^24^,^25^ indicate that force-induced unfolding of the bundles is required to unmask the VBSs. Thus, it is hypothesised that force-induced conformational changes in talin lead to the recruitment of vinculin and the stabilization of FAs. However, to what extent such mechanisms operate in cells is unclear.

Structure–function studies on talin have been hampered by the fact that (i) most cell types express two structurally and functionally related talin isoforms^26^,^27^, (ii) knockout or knockdown of talin1 leads to upregulation of talin2^4^ and (iii) knockdown leaves residual talin^4^,^28^, complicating the interpretation of results.

Here, we use newly derived talin1 and talin2 knockout (TKO) cells that grow in suspension, and only adhere to ECM, spread and assemble FAs following expression of functional talin constructs. By reconstituting TKO cells with structure-based talin point and deletion mutants, we define the role of specific talin and vinculin domains in engaging the actomyosin machinery, the assembly and stability of FAs, and the establishment of cell polarity. We demonstrate that vinculin binding within domains R2R3 acts to ‘unlock' ABS2 of talin, and that this is regulated either by prior activation of vinculin, or by the application of force across talin provided by actin-binding to ABS3. These findings demonstrate how talin, vinculin and actin interact with one another to form the major mechanosensory module of the FA.

## Results

### Talin regulates FA size and cell polarity

**Figure 1:**
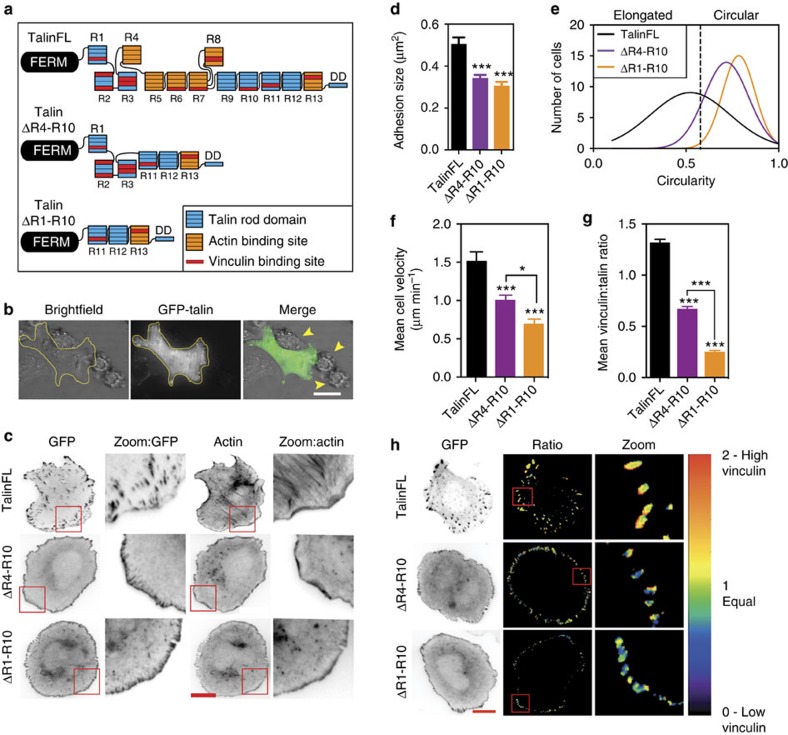
Talin rod domains regulate cell polarity and migration, FA morphology and FA composition. (**a**) Cartoon of talin constructs expressed as N-terminally tagged GFP-fusion proteins. The talin FERM domain is linked to the flexible talin rod which consists of 13 helical bundles (R1-R13) terminating in a dimerization helix (DD). Constructs in which the R4-R10 (talΔR4-R10) and R1-R10 (talΔR1-R10) domains have been deleted are shown. Colours indicate binding sites for actin (orange) and vinculin (red). (**b**) Talin1 and talin2 knockout (TKO) cells cells transfected with talinFL, 24 h after plating on fibronectin; arrows indicate non-transfected cells, which do not spread. (**c**) TKO cells expressing indicated GFP-talin fusion constructs were plated on fibronectin and stained for F-actin. Magnified regions are from the area framed in red. Note the colocalisation of talΔR1-R10 or talΔR4-R10 with F-actin at the cell edge. Scale bars, 10 μm. (**d**) Quantification of FA size in TKO cells expressing indicated constructs. Note that talΔR1-R10 or talΔR4-R10 have smaller FAs compared with cells expressing talinFL (*n*>70 cells, from three independent experiments, error bars are ±s.e.m., ***=*P*<0.001 (ANOVA)). (**e**) Quantification of cell circularity of cells expressing GFP-talin constructs. Note that cells expressing talΔR1-R10 or talΔR4-R10 are more circular than cells expressing talinFL (*n*=90 cells, from three independent experiments). (**f**) TKO cells expressing the three GFP-talin constructs were tracked over 24 h. Both talΔR1-R10 and talΔR4-R10 cells have significantly reduced velocity compared with talinFL cells; talΔR1-R10 cells are slower than talΔR4-R10 cells (*n*=20 cells, from two independent experiments, error bars are±s.e.m., *=*P*<0.05; ***=*P*<0.001 (ANOVA). (**g**,**h**) Ratiometric imaging was used to determine the proportion of vinculin present at adhesions in TKO cells expressing indicated constructs. Quantitative analysis (in **g**) shows that vinculin levels are reduced in adhesions in talin rod deletion mutants. Cells expressing the talΔR1-R10 construct (the largest deletion) had the lowest levels of vinculin in adhesion complexes. Scale bar in **h**, 10 μm. (*n*>65 cells, from three independent experiments, error bars are±s.e.m., ***=*P*<0.001 (ANOVA) compared with talinFL unless where indicated).

To investigate how the 11 potential VBSs in the talin rod^12^ contribute to FA formation, we deleted rod domains containing either 4 (talΔR4-R10) or 9 (talΔR1-R10) of the VBSs (Fig. 1a; Supplementary Fig. 1b). These constructs were tagged with GFP and expressed in TKO cells, which lack endogenous talin (Supplementary Fig. 1a) and do not adhere to ECM and do not spread (Fig. 1b; Supplementary Movie 1)^4^. Expression of GFP-tagged full-length talin (talinFL) rescued integrin activation (Supplementary Fig. 1c), cell adhesion, spreading and FA formation (Fig. 1b,c), and cells displayed both small peripheral adhesions and mature FAs connected to prominent actin stress fibres. In contrast, although the talin rod deletion mutants rescued integrin activation and spreading, the cells lacked large FAs (Fig. 1c; Supplementary Fig. 1c). TalΔR1-R10 cells displayed mostly small peripheral adhesions, and while talΔR4-R10 cells assembled slightly larger structures (Fig. 1d), both generally lacked actin stress fibres, although adhesions were connected to thin actin filaments (Fig. 1c; Supplementary Fig. 2a,b). Moreover, cells expressing these mutants were largely unpolarized (Fig. 1e), and cell motility was reduced compared with talinFL cells (Fig. 1f); cell polarization is a prerequisite for directional migration.

Vinculin, which binds talin and actin, is important for full engagement of adhesion complexes with the force machinery^8^,^16^. Thus, the small peripheral adhesions exhibited by talΔR1-R10 and talΔR4-R10 cells might result from reduced vinculin-binding. Indeed, we observed a twofold reduction in the vinculin/talin ratio in talΔR4-R10 cells, and a fivefold reduction in talΔR1-R10 cells compared with talinFL cells (Fig. 1g,h). We conclude that both talin R1-R3 and R4-R10 contain functional VBSs that recruit vinculin to FAs. The presence of vinculin in talin ΔR1-R10 adhesions likely indicates that the two VBSs in R11-R13 are also functional, as proposed by others^28^,^29^.

### Talin domains R1-R3 contain key vinculin binding sites

**Figure 2:**
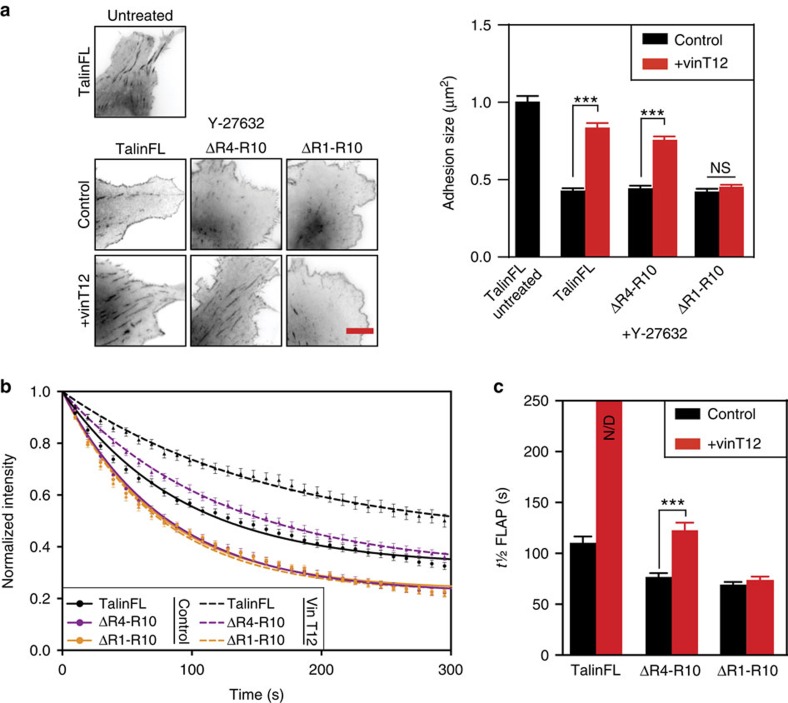
Vinculin regulates talin by binding to R1-R3 domains. (**a**) NIH3T3 cells expressing talinFL, talΔR1-R10 or talΔR4-R10 with vinFL (control) or vinT12 were treated with DMEM containing 50 μM Y-27632 or an equivalent volume of water for 30 min before fixation. The effect on adhesions was analysed assessing the signal from the expressed GFP-talin fusion constructs. FAs in control cells expressing talinFL are dramatically reduced in size following treatment with Y-27632. Note that talinFL and talΔR4-R10, but not talΔR1-R10, in FAs are stabilised by co-expression of vinT12 (*n*=15 cells, representative of three independent experiments, error bars are±s.e.m., ***=*P*<0.001 (ANOVA)); scale bar, 5 μm. (**b**) Curves from fluorescence loss after photoactivation (FLAP) experiments in NIH3T3 cells expressing either talinFL, talΔR4-R10 or talΔR1-R10 with and without vinT12. (**c**) Quantification of half-time of FLAP (t½ FLAP) to assess the mobility of talin constructs in FAs. Note that vinT12 reduces the mobility (increase of t½) of talinFL and talΔR4-R10 but not talΔR1-R10. The almost linear decay of talinFL in the presence of vinT12 prevented appropriate fitting, hence accurate t½ FLAP could not be determined; N/D, not determined (*n*=25–66 FAs, from three independent experiments, error bars are±s.e.m., ***=*P*<0.001 (ANOVA)).

Inhibition of actomyosin-mediated tension with Rho Kinase (ROCK) or myosin inhibitors normally results in FA disassembly^16^. By binding to talin, constitutively active full-length vinculin (vinT12) stabilises FAs which become insensitive to such drugs^7^,^16^. To investigate which talin rod domains are key to this effect, talinFL or the talin deletion mutants were co-expressed with either full-length vinculin (vinFL) or vinT12, and the cells treated with Y-27632. As the talin deletion mutants do not support FA maturation in TKO cells, we used NIH3T3 cells for these experiments (endogenous talin supports FA formation which become GFP-talin positive; Supplementary Fig. 3a). As expected, Y-27632 reduced the size of GFP-talinFL positive FAs while vinT12 stabilized FAs (Fig. 2a). The intensity of GFP-talinFL in FAs also decreased more than in cells co-expressing vinT12 (Supplementary Fig. 4a). Interestingly, while vinT12 also stabilized talΔR4-R10 in FAs to a significant degree, it had no effect on talΔR1-R10 (Fig. 2a and Supplementary Fig. 4a), suggesting that binding of vinculin to the R1-R3 region of the talin rod is particularly important for adhesion stability.

Vinculin-induced FA stabilization goes alongside reduced talin turnover^7^,^30^. To investigate the turnover of talinFL and the deletion mutants, we used talin constructs tagged with photoactivatable-GFP (PAGFP) and assessed fluorescence loss after photoactivation (FLAP; for details see Supplementary Fig. 3b and Supplementary Movie 2). The results showed that the rates of turnover of talΔR4-R10 and talΔR1-R10 were significantly faster than talinFL (Fig. 2b,c; Supplementary Fig. 3c; Supplementary Movie 3). Co-expressing vinT12 significantly decreased turnover of talinFL, and to some extent talΔR4-R10, but not the talΔR1-R10 mutant (Fig. 2b,c; Supplementary Fig. 3c). These results were confirmed using FRAP (Supplementary Fig. 3d). To establish that the inhibitory effect of vinT12 on talin dynamics was due to a direct interaction, we used a vinT12^A50I^ mutant with reduced talin binding. This construct had no effect on talinFL turnover as assessed by FLAP (Supplementary Fig. 3e and Supplementary Movie 4). These results clearly indicate that binding of active vinculin to the R1-R3 region of the talin rod plays an important role in stabilizing FAs.

### ΔR2R3 suppresses FA dynamics independently of vinculin

**Figure 3:**
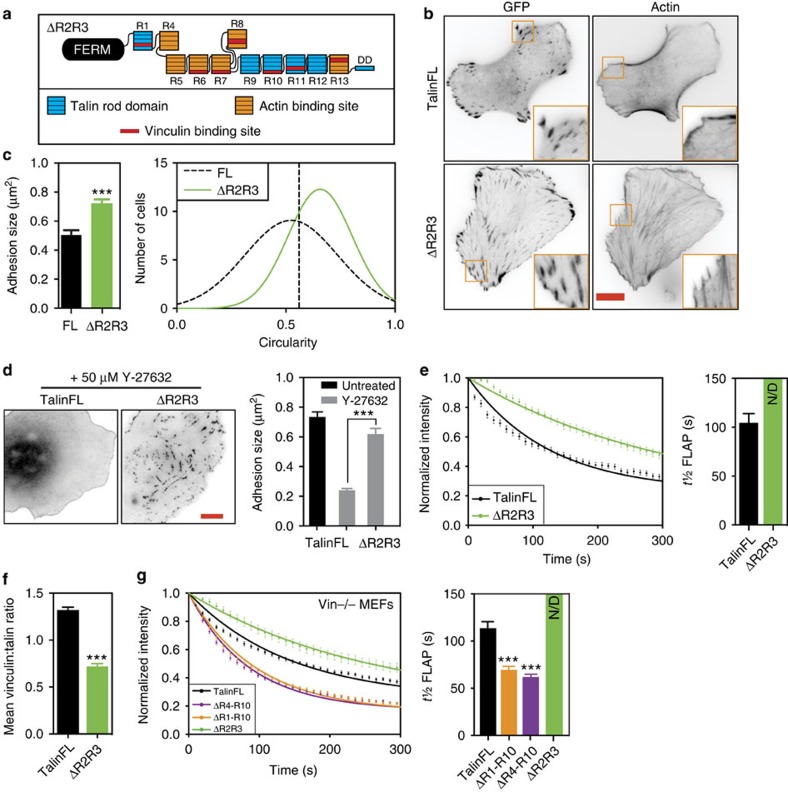
Deletion of Talin R2R3 stabilises FAs. (**a**) Cartoon of the talΔR2R3 construct. (**b**,**c**) TKO cells expressing talΔR2R3 have more prominent actin stress fibres associated with larger FAs than those in cells expressing talinFL; cells expressing talΔR2R3 are more circular than talinFL cells (*n*=70–90 cells, from three independent experiments, error bars are±s.e.m., ***=*P*<0.001 (ANOVA)), scale bar in **b**, 10 μm. (**d**) Left panel, representative images of TKO cells expressing indicated constructs treated with DMEM containing 50 μM Y-27632 or an equivalent volume of water for 30 min. Note that Y-27632 resulted in a reduction of adhesion size of cells expressing talinFL compared with either untreated control cells or cells expressing talΔR2R3. (**e**) FLAP measurement curves of talΔR2R3 and talinFL in TKO cells. Curves (left panel) and quantification of *t*½ (right panel) show the reduced mobility of talΔR2R3 in comparison to talinFL (*n*=49–51 FAs, from three independent experiments). The almost linear decay of talΔR2R3 prevented appropriate fitting, hence accurate *t*½ FLAP could not be determined; N/D, not determined. (**f**) Quantification of vinculin recruitment in TKO cells expressing talΔR2R3. Note the reduced levels of vinculin in FAs of talΔR2R3 cells compared with levels in FAs of control cells expressing talinFL (*n*>70, from three independent experiments, error bars are±s.e.m., ***=*P*<0.001 (ANOVA)). (**g**) FLAP curves of experiments in MEFvin^−/−^ cells (left panel) transfected with talinFL or talΔR2R3. Quantification of FLAP (right panel) showing t½ times for each construct. Note that even in absence of vinculin, talΔR2R3 has a slower turnover than talinFL. The almost linear decay of talΔR2R3 prevented appropriate fitting, hence accurate *t*½ FLAP could not be determined; N/D=not determined. (*n*=35–60 FAs, from three independent experiments, error bars are±s.e.m.).

Four out of the five VBSs in talin R1-R3 are in R2R3 (Fig. 1a). We therefore expected that TKO cells expressing a talΔR2R3 construct (Fig. 3a) would have smaller and less stable FAs. To our surprise, they were even larger than FAs in talinFL cells, although the cells were more rounded (Fig. 3b,c). All FAs in talΔR2R3 cells were linked to prominent actin stress fibres (Fig. 3b), and strikingly, the FAs remained stable when treated with Y-27632 (Fig. 3d and Supplementary Fig. 4b). Comparable results were seen in NIH3T3 cells (Supplementary Fig. 4c). Co-expression of vinT12 with talΔR2R3 caused no further increase in FA stability (Supplementary Fig. 4d). Moreover, FLAP experiments showed that talΔR2R3 turnover was greatly diminished compared with talinFL (Fig. 3e and Supplementary Movie 5). As expected, FAs in talΔR2R3 cells had a significantly reduced vinculin to talin ratio compared with control cells (Fig. 3f and Supplementary Fig. 5). Furthermore, talΔR2R3 exhibited reduced turnover even when expressed in vinculin null cells (Fig. 3g). Clearly, the stabilizing effect induced by deleting talin R2R3 is vinculin independent.

### A role for talin ABS2 in FA dynamics

**Figure 4:**
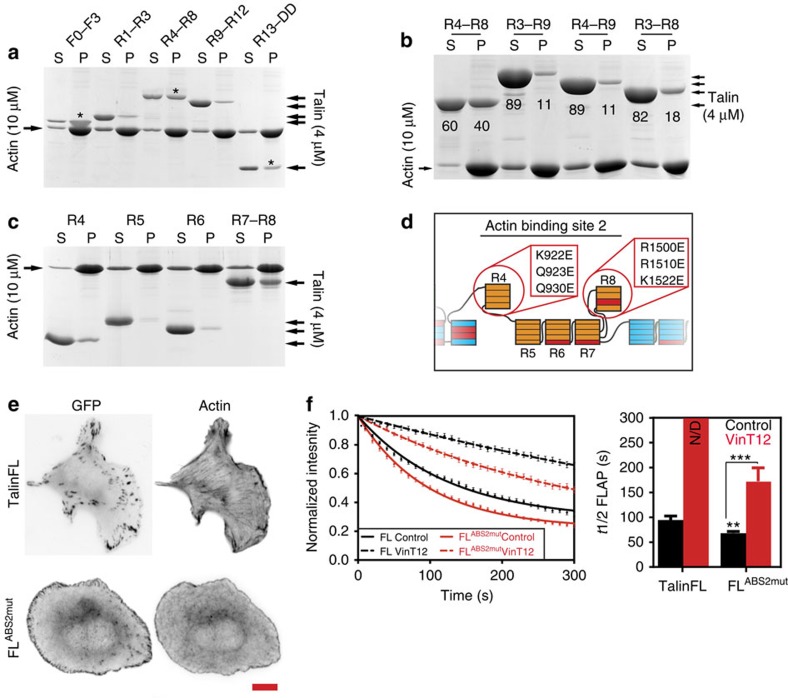
ABS2 is a key regulator of talin. (**a**,**b**) Recombinant talin polypeptides were incubated with F-actin, the actin pelleted, and supernatants (S) and pellets (P) analysed by SDS–PAGE. (**a**) Talin FERM domain F0-F3 (ABS1) followed by talin rod domains R1-R3, R4-R8 (ABS2), R9-R12 and R13-DD (ABS3). Asterisks show the domains containing the known ABSs. (**b**) Actin co-sedimentation assays show that actin binding to ABS2 is reduced when either domain R3 or R9 is present; numbers show percent band density (**c**) Actin co-sedimentation studies using the 4 sub-domains that make up ABS2; Note that R4 and R7-R8 bind actin. (**d**) Scheme outlining point mutations in the talin rod that reduce actin binding to ABS2. (**e**) Expression of talin ABS2 mutant (FL^ABS2mut^) in TKO cells rescued cell spreading, but resulted in rounder cells with smaller FAs compared with talinFL cells (see also Supplementary Fig. 6a,b; scale bar, 10 μm). (**f**) FLAP experiments in TKO cells show that the turnover of talinFL^ABS2mut^ is increased compared with talinFL (FL control). Note that co-expression with vinT12 reduced the turnover of talinFL^ABS2mut^ to a lesser extent than talinFL. The almost linear decay of the talinFL in presence of vinT12 (FL VinT12) prevented appropriate fitting, hence accurate *t*½ FLAP could not be determined; N/D=not determined (*n*=28–56 FAs, from three independent experiments, error bars are±s.e.m., ***=*P*<0.001 (ANOVA)).

One possibility is that deletion of R2R3 may activate the adjacent actin-binding site (ABS2) originally mapped to residues 951–1327 (ref. 14) (roughly equivalent to R4-R6). However, R7R8 has also now been shown to bind F-actin^21^. To map ABS2 more precisely, we expressed several new talin rod fragments designed according to domain boundaries. Talin R4-R8 co-sedimented with F-actin (Fig. 4a) to about the same extent as ABS1 in the talin head and ABS3 in the C-terminal R13 rod domain. Interestingly, R1-R3 and R9-R12 also co-sedimented with F-actin, though to a lesser degree, indicating that like filamin^31^, talin interacts with actin at several sites distributed along the length of the molecule.

To explore the possibility that domains flanking ABS2 might influence its activity, we designed ABS2 constructs containing the adjacent R3 and R9 domains. Interestingly, inclusion of these domains either together (R3-R9) or individually (R4-R9 or R3-R8) actually reduced actin binding to ABS2 (Fig. 4b). These results indicate that domains flanking ABS2 suppress its activity, and suggest that conformational changes in these domains have the potential to activate ABS2.

To identify the major actin binding determinants in R4-R8, we examined the ability of individual domains to bind F-actin (Fig. 4c). Both R4 and the R7R8 double domain bound F-actin (the latter is consistent with previous results^21^). In contrast, R5 and R6 bound only weakly if at all to F-actin, and previous studies have shown that R7 alone does not bind actin^21^. The results indicate that R4 and R8 are the key determinants of ABS2, and perhaps bind cooperatively to F-actin. Both domains have anomalously high pI values (9.5 and 7.8, respectively) compared with an average pI of 5.4 for the talin rod, and will be positively charged at physiological pH as required for binding to the negatively charged surface of F-actin. High pI values are also observed for ABS1 and ABS3 (Supplementary Fig. 6).

Analysis of the distribution of conserved positively charged and hydrophobic amino acids in the R4 and R8 domain structures highlights a number of residues that may contribute to actin binding. By serially mutating these residues (Fig. 4d), a R4-R8 construct with reduced ability to bind F-actin (∼60% inhibition) was generated (B.Goult and M.Schwartz personal communication). A GFP-tagged talinFL^ABS2mut^ containing these mutations rescued cell spreading in TKO cells, but the FAs were smaller and the number of actin stress fibres markedly reduced compared with talinFL cells (Fig. 4e; for quantification Supplementary Fig. 7). Furthermore, FLAP experiments showed that turnover of talinFL^ABS2mut^ was significantly faster than talinFL and similar results were obtained by deleting the R8 domain (Fig. 4f and Supplementary Movie 7, Supplementary Fig. 7c and Supplementary Movie 6). Both talinFL and talinFL^ABS2mut^ were stabilized by co-expression of vinT12; however, it had a less pronounced effect on talinFL^ABS2mut^. Thus, ABS2 is an important factor determining talin turnover in FAs.

### ABS2-actin and vinculin-actin regulate talin dynamics

**Figure 5:**
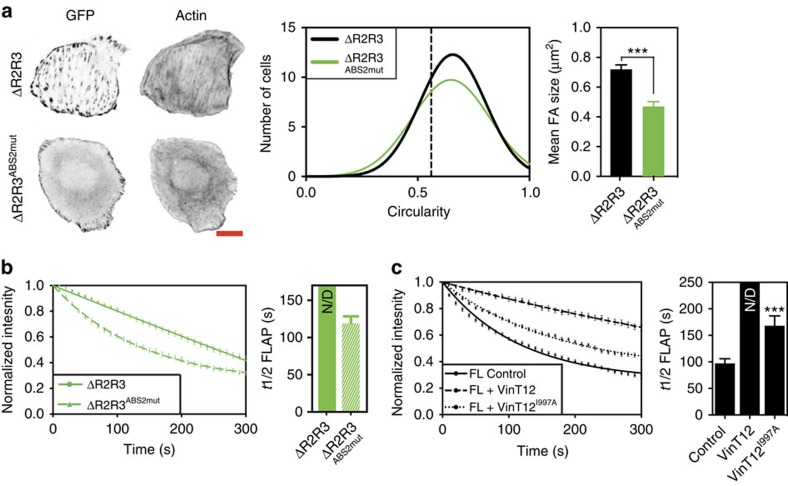
Talin R2R3 regulate the availability of ABS2 for actin binding. (**a**) The talin ABS2 point mutations were introduced into talΔR2R3 and this construct (talΔR2R3^ABS2mut^) was expressed in TKO cells. Scale bar, 10 μm. TalΔR2R3^ABS2mut^ cells had a similar circularity to talΔR2R3 cells, but lacked prominent actin stress fibres and had smaller FAs than talΔR2R3 cells (*n*>70 cells, from three independent experiments, error bars are±s.e.m., ***=*P*<0.001 (*t*-test)). (**b**) FLAP experiments show that talΔR2R3^ABS2mut^ had a faster turnover than talΔR2R3 (*n*=48–51 FAs, from three independent experiments, error bars are±s.e.m.). (**c**) FLAP experiments of talinFL co-expressed with either vinT12 or a mutant form of vinT12 with reduced actin binding (vinT12^I997A^). Note that mutating the actin-binding site within the vinculin tail reduces the stabilizing effect of vinT12 (*n*=36–47 FAs, from three independent experiments, error bars are±s.e.m.).

To test whether the stabilizing effects of talΔR2R3 are dependent on ABS2, we expressed a talΔR2R3^ABS2mut^ mutant in TKO cells. The construct rescued cell spreading, but the cells had much smaller FAs and lacked the prominent F-actin stress fibres seen with the talΔR2R3 construct (Fig. 5a). Furthermore, using FLAP, we found that the talΔR2R3^ABS2mut^ had a significantly faster turnover than talΔR2R3 (Fig. 5b and Supplementary Movie 8) demonstrating that the stabilizing effects of talΔR2R3 are indeed partly mediated through ABS2.

An additional factor influencing FA dynamics, migration and traction force is the ability of vinculin to bind actin via its C-terminal tail^32^,^33^. To establish whether this is also a factor regulating talinFL turnover, we used a vinT12^I997A^ mutant with reduced actin-binding and bundling activity^8^. The vinT12^I997A^ construct still produced a significant reduction in the turnover of talinFL, but the reduction was not as great as with vinT12 (Fig. 5c and Supplementary Movie 9). Altogether, these experiments strongly suggest that actin binding to talin ABS2 contributes to FA stabilization, and that this is regulated by vinculin binding to R2R3. However, the ability of vinculin to bind directly to both talin and F-actin also plays a significant role.

### Talin-actin link is required for traction force generation

**Figure 6:**
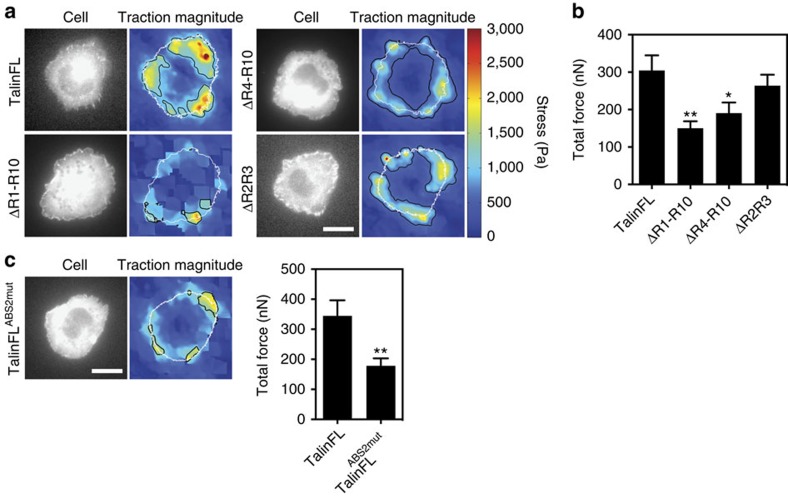
The link between talin ABS2 and F-actin is critical for force exertion. (**a**) Representative images from traction force microscopy (TFM) experiments. Colour spectrum indicates stress magnitude (Pa), with areas of low traction in blue and high traction in red. (**b**) Quantification of total force (nN) from TFM experiments shows that TKO cells expressing constructs lacking ABS2 (talΔR1-R10 and talΔR4-R10) exert less force than cells expressing talinFL (*n*=26–46 cells, error bars are±s.e.m., *=*P*<0.05, **=*P*<0.01 compared with talinFL (ANOVA)). (**c**) TFM experiments were repeated with the talinFL^ABS2mut^ construct. Cells expressing talinFL^ABS2mut^ had reduced force exertion compared with cells expressing talinFL (*n*=21–30 cells, error bars are±s.e.m., **=*P*<0.01 (*t*-test)). Scale bars in **a** and **c**, 10 μm.

The link between the adhesion plaque and actomyosin is essential to generate the forces required for cell migration. To investigate the role of talin in this process, we used TKO cells expressing various talin mutants, and traction force microscopy to examine force transmission to substrate. The largest traction stresses detected were at the cell periphery, where the majority of adhesion complexes are localized (Fig. 6). Highest traction stresses were observed in talinFL and talΔR2R3 cells, while cells expressing talin ABS2 deletion or point mutants exhibited significantly reduced force transmission (Fig. 6a,b). Quantification showed that cells expressing talΔR2R3 were able to exert similar forces on the ECM to cells expressing talinFL (Fig. 6a,b). In contrast, cells expressing talΔR4-R10 or talΔR1-R10, which lack ABS2 and several VBSs, showed a 45 and 55% reduction respectively in the force transmitted to ECM (Fig. 6a,b). Similarly, TKO cells expressing talinFL^ABS2mut^ displayed a 50% weaker force exertion than cells expressing talinFL (Fig. 6c). These data clearly demonstrate that ABS2 plays a key role in actomyosin-mediated force transmission.

### Talin ABS3 is required for FA initiation and cell spreading

**Figure 7:**
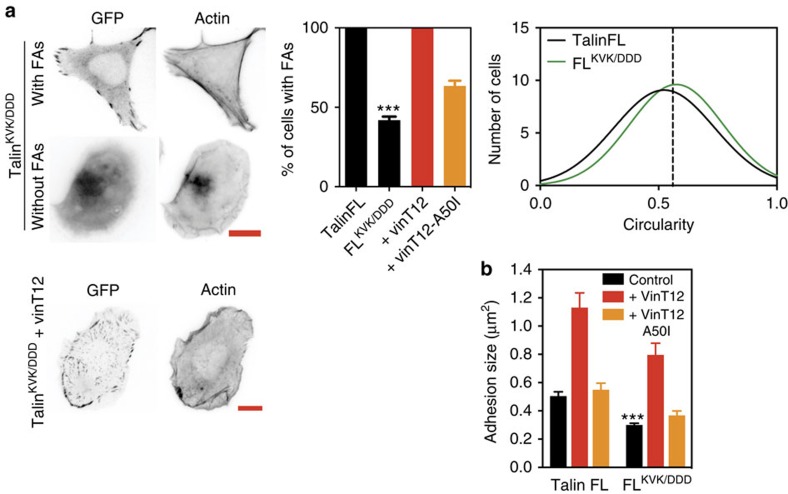
The role of talin ABS3 in FA assembly can be bypassed by active vinculin. (**a**) While expression of talinFL^KVK/DDD^ in TKO cells rescues cell spreading,<50% of the cells display FAs connected with F-actin. FA formation in all cells was rescued by co-expression with vinT12 but not by a vinT12^A50I^ mutant with reduced talin binding. Scale bars, 10 μm. (*n*>70 cells, ***=*P*<0.001 (*t*-test), error bars are±s.e.m.). (**b**) In those talinFL^KVK/DDD^ cells that do have FAs, the FAs are smaller compared with those in talinFL cells. Co-expression with vinT12 (but not vinT12^A50I^) rescued FA size in cells expressing talinFL^KVK/DDD^ (*n*>70 cells, ***=*P*<0.001 (ANOVA), error bars are±s.e.m.).

While the above experiments demonstrate a key role for ABS2 in FA maturation and engagement with the actomyosin machinery, the role of ABS3 has remained unclear. One hypothesis, largely derived from *in vitro* experiments, is that ABS3 might support the initial force transduction that is required to unravel the R2R3 helical bundles, and promote vinculin binding. If this were the case, one would expect that abolishing actin binding to ABS3 would result in a failure to form stable adhesions. To test this hypothesis, we used a talinFL^KVK/DDD^ mutation that reduces actin binding to ABS3 by >70%, without affecting dimerization which is essential for actin binding^22^. Interestingly, while the GFP-tagged talinFL^KVK/DDD^ mutant rescued TKO cell spreading, ∼45% of cells lacked FAs (Fig. 7a,b). Moreover, in the majority of cells, GFP-talinFL^KVK/DDD^ showed a diffuse cytoplasmic distribution with no clear localization to actin filaments, unlike constructs with an intact ABS3. These experiments suggest that F-actin binding to ABS3 supports the force required for the initial events leading to FA formation.

However, about half of talinFL^KVK/DDD^ cells contained some FAs (Fig. 7a,b). These cells exerted weaker traction forces, migrated slower and contained less vinculin in FAs compared with cells expressing talinFL (Supplementary Fig. 8). However, the fact that they had clearly visible FAs suggested that FA initiation via ABS3 can be bypassed. We therefore tested whether activated vinculin, which in turn activates ABS2, and can itself bind directly to F-actin, might bypass ABS3. Indeed, we found that co-expression of vinT12 with the talinFL^KVK/DDD^ mutant was able to fully rescue FA formation, unlike co-expression of vinT12^A50I^ (Fig. 7a,b). Together, our data demonstrate that ABS3 plays a key role in the initiation of FA assembly, but that its role can be bypassed by pathways that activate vinculin and allow the association between vinculin and talin.

## Discussion

The development of FAs involves (i) formation of small, dot-like nascent adhesions independent of actomyosin-mediated tension and (ii) their tension-dependent maturation into FAs^34^,^35^,^36^. Talin is seen early in adhesion complex assembly^37^, binds and activates integrins^38^,^39^ and regulates the recruitment of other proteins including vinculin, paxillin and FAK to FAs^4^,^28^,^40^. Using TKO cells, we demonstrate that talin is indeed essential for integrin activation, cell adhesion and spreading, and is at the core of adhesion complex assembly, the regulation of adhesion strength and engagement with actin.

TKO cells expressing a talin ABS3 mutant (talinFL^KVK/DDD^)^22^ exhibit a marked reduction in assembly of adhesion clusters suggesting that in the absence of ABS3-mediated coupling to actomyosin, the force-dependent conformational changes in talin required to expose cryptic VBSs and to activate ABS2 are inoperative. As a result, adhesion complexes that do form are unstable. ABS3 may also be involved in the correct localization of talin, bringing it into position to bind and activate integrins and initiate adhesion complex assembly^41^. Such a model is suggested by our finding that the talinFL^KVK/DDD^ mutant showed little enrichment in actin-rich lamellipodia (Fig. 7a) compared with constructs with an intact ABS3 (Fig. 1c). However, the role of talin ABS3 in adhesion assembly can be bypassed by constitutively active vinculin, suggesting that pathways that activate endogenous vinculin may also drive complex assembly. Consistent with this, the muscle-detachment phenotype caused by expressing a talin ABS3 mutant during Drosophila embryogenesis was partially rescued by vinculin^42^.

To date, the role of ABS2 in the central part of the talin rod has been ignored. Here we establish that talin ABS2 (R4-R8) is essential for stabilization of FA complexes, and for the generation of maximal traction force (Figs 4 and 6). Our biochemical experiments indicate that domains flanking ABS2 suppress its activity (Fig. 4c), suggesting that talin must undergo a conformational change that relieves this inhibitory effect. Since the talinFL^KVK/DDD^ mutation inhibits FA assembly (Fig. 7a), it seems likely that activation of ABS2 is initially regulated by engagement of ABS3 with actomyosin. However, the fact that the talinFL^KVK/DDD^ phenotype is rescued by vinT12 suggests additional regulation of ABS2 by vinculin binding to R3 (Fig. 7a, b). Indeed, our experiments with talin rod deletion mutants show that the VBSs in the R1-R3 domains are particularly important for FA stabilization, and magnetic tweezer experiments demonstrate that (i) force unmasks the VBSs in R2R3 (ref. 16) and (ii) that R3 is the initial mechanosensing domain unfolding at ∼5 pN (refs 24, 43). This conclusion is consistent with structural data which shows that while R2 is stabilized by R1 (ref. 10), R3 is the least stable of the three domains because of the presence of four threonine residues buried within its hydrophobic core^10^,^24^.

Based on these results, we postulate that the initial association between talin ABS3 and actin provides sufficient force to overcome the 5 pN threshold to unfold the talin R3 helical bundle. This allows vinculin to bind to R3, relieving the inhibitory effect of R3 on ABS2 which then binds F-actin, reinforcing the link with the actomyosin machinery, leading to adhesion maturation. When the initial force acting on talin ABS3 is insufficient to unfold the R3 domain, binding of vinculin is suppressed, ABS2 is not activated and the complex disassembles, as seen in short-lived nascent adhesions^36^.

Ratiometric imaging with talin deletion mutants and vinculin (Figs 3f and 1h) shows that the R2R3, R4-R10 and the C-terminal R11-R13 talin rod domains can all bind vinculin *in vivo*, confirming *in vitro* data of multiple VBSs along the talin rod^12^. Data demonstrating that activated vinculin stabilizes talin mutants containing talin R2R3 and R4-R10 domains in FAs, and the FA structure itself (Fig. 2a,b) indicates that vinculin drives adhesion assembly through binding to VBSs in these domains. Vinculin-mediated adhesion stabilization depends in part on the actin binding activity of vinculin itself since maximal stability was not achieved using a constitutively active form of vinculin containing a mutation in its own actin binding site (Fig. 5c). These observations, together with the previously reported force mediated recruitment of vinculin to FAs^16^,^25^,^44^, and the increased ratio of vinculin/talin during FA maturation^45^, suggest that a gradual increase of force induces vinculin to bind to different sites in talin, thus strengthening the connectivity between the adhesion complex and the cytoskeleton. This additional mode of regulation through vinculin, whose own activity can be regulated by actomyosin-mediated forces, adds a regulatory module which appears key for the cell to respond to changes in the mechanical environment^16^.

**Figure 8:**
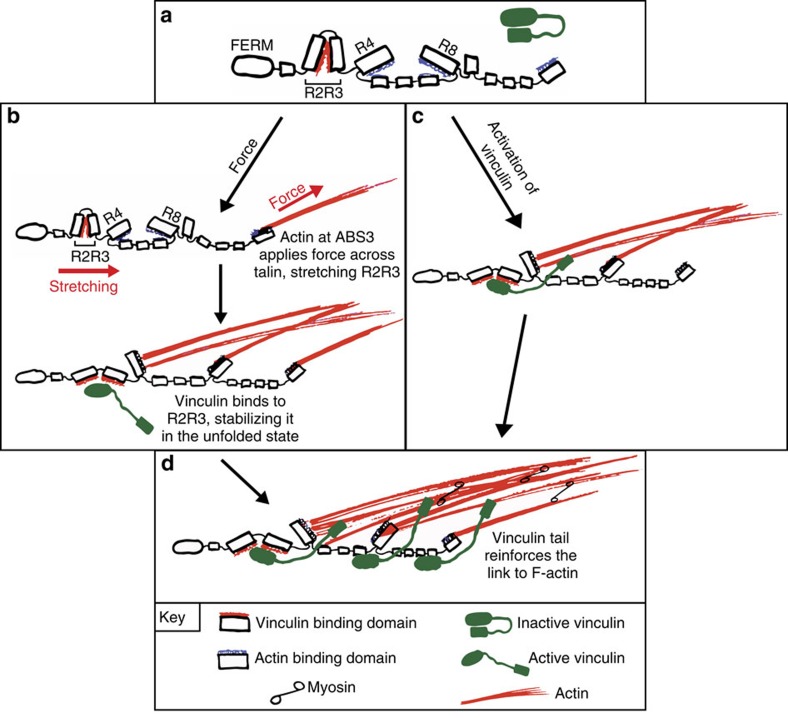
Model for vinculin supported talin engagement with the actomyosin machinery. (**a**) Talin in which the vinculin and actin binding sites in R2R3 and R4-R8 (ABS2), respectively are cryptic. Inactive vinculin in green. (**b**,**c**) Two possible mechanisms for linking talin with the actomyosin machinery; (**b**) force-dependent pathway: actin binding to ABS3 leads to force exertion across talin, stretching R2R3 and unmasking its previously cryptic vinculin binding sites. A combination of force and vinculin binding to R2R3 also relieves the inhibitory effect of R3 on ABS2, allowing actin binding. (**c**) Vinculin driven pathway: binding of activated vinculin to talin R2R3 domains unlocks ABS2, allowing actin to bind to talin independent of ABS3. (**d**) Full engagement with actin occurs through actin binding of ABS2 and ABS3, with vinculin stabilizing the link between talin and actin.

We propose the following two-step model for talin activation and adhesion maturation which is key for co-ordinated force transduction and polarized cell migration (Fig. 8). Initially, talin, the bulk of which exists in an autoinhibited form in the cytosol^46^,^47^, is recruited to the plasma membrane, the actin-rich lamellipodium and adhesion sites through pathways involving actin binding to ABS3, or its interaction with RIAM, integrins, PIP_2_, exosomes^48^ and FAK^49^,^50^,^51^,^52^. Whether kindlin, which appears to precede talin in nascent adhesions^45^, is involved in recruiting talin is unclear. At the leading edge, the talin head-rod auto-inhibitory interaction is disrupted possibly by PIP2 (ref. 50), and talin synergises with kindlins to activate integrins^9^ leading to the formation of nascent adhesions. Subsequently, actomyosin-mediated tension across talin, bound to integrins at one end and actin via ABS3 at the other, leads to unfolding of the R3 helical bundle relieving inhibition of ABS2 and exposing the two high affinity VBSs in R3, which can bind vinculin^16^,^24^. Vinculin may prevent refolding of R3 locking ABS2 into an active state. The combined link of talin ABS2 and ABS3 to F-actin and the ability of vinculin to cross-link talin to actin stabilises talin in the adhesion complex. This hypothesis is supported by the recently published findings that talin engaged with F-actin at FAs is under tension, and is stretched and positioned by this F-actin linkage, thus orchestrating the molecular architecture of the FA^53^. Further increases in myosin-II mediated force generation may subsequently activate additional VBSs localized along the talin rod leading to recruitment of more vinculin molecules, resulting in the maximal stabilization of the overall FA structure. The fact that vinT12 can bypass the role of talin ABS3 in FA assembly, suggests that pathways that activate vinculin (for example, via PIP2 (refs 54, 55)) may also drive FA assembly. FAs then act as signalling hubs controlling many aspects of cell behaviour including polarity and migration.

## Methods

### Cell lines and transfections

NIH3T3 fibroblasts and vinculin^−/−^ mouse embryonic fibroblasts (MEFs) were cultured in Dulbecco's modified Eagle's medium (DMEM) supplemented with 10% FCS, 1% L-glutamine and 1% non-essential amino acids. For the generation of talin1 and talin2 knock out cells, cells were isolated from the collecting ducts of 5–6-weeks-old talin1^flox/flox^ mice/ talin2 null mice^56^ as described by Husted *et al*.^57^ and immortalized with pSV40 plasmid. Loci for talin1 gene in collecting duct cells were deleted with adenovirus expressing Cre recombinase. Multiple clones of the double null cells were prepared by performing serial dilution cloning and verified similar functions in different clones. Gene deletion was verified by both PCR and immunoblotting for talin 1 and talin 2 (Supplementary Fig. 1a). Talin null cells were cultured in DMEM F-12 supplemented with 10% FCS, 1% L-glutamine, 15 μM HEPES and 1% Antimycotic solution (Sigma). All cells were cultured at 37 °C with 5% CO_2_. Transient transfections were performed using Lipofectamine and Plus reagents (Life Technologies) as per the manufacturer's instructions.

### Antibodies, reagents and plasmids

Bovine fibronectin was purchased from Sigma and used at 10 μg ml^−1^ diluted in PBS (Sigma). Y-27632 dihydrochloride (Tocris Bioscience) was dissolved in water and used at 50 μM for 30 min. Mouse anti-vinculin antibody (clone hVin1) (Sigma, UK) was diluted (1:500) in 1% Bovine Serum Albumin (BSA) (cat: V9131, Sigma, UK). Dylight 594-conjugated AffiniPure Donkey Anti-Mouse IgG (cat: 715-585-150, Jackson ImmunoResearch, USA) was used as a secondary antibody, diluted in 1% BSA (1:500). Site-directed mutagenesis was performed using the QuikChange Lightning Multi Site-Directed Mutagenesis kit (Agilent, USA). For western blotting, the primary antibodies used were mouse anti-talin (8d4) (cat: T3287, Sigma) and mouse anti-GFP (cat: 11 814 460 001, Roche), diluted 1:500 and 1:250, respectively, in 2% milk (PBS 0.1% Tween). The secondary antibody was goat anti-mouse IgG conjugated to horseradish peroxidase (cat: A9917, Sigma), diluted 1:5,000 in 2% milk (PBS 0.1% Tween). Bands were detected using enhanced luminol-based chemiluminescent substrate (Promega).

### Western blots

Cells were transfected with the desired talin constructs, incubated for 24 h before lysis with Laemmli buffer. Samples were run on an SDS–polyacrylamide gel electrophoresis (SDS–PAGE) gel, 90 v, 15 min followed by 120 v, 1 h. Proteins were transferred to a nitrocellulose membrane at 250 mAmps, 2 h. The membrane was blocked for 1 h with 5% milk (PBS 0.1% Tween) and probed with the indicated primary antibodies. Uncropped scans of the blots are shown in Supplementary Fig. 9.

### Immunofluorescence and microscopy

Cells transfected with GFP- and/or mCh-tagged proteins were incubated overnight on glass bottom dishes (MatTek), fixed with 4% paraformaldehyde and permeabilised with 0.5% Triton X-100 (Sigma). Samples were incubated with the primary antibody for 60 min, and then washed thrice with PBS. Secondary antibody staining followed the same procedure. Fixed samples were imaged using a Delta Vision RT microscope (Applied Precision) equipped with a 60 × /1.42 Plan Apo oil immersion objective (Zeiss). Images were acquired with a CoolSnap HQ camera (Photometrics).

### Image analysis and ratio imaging

Image analysis was carried out using Fiji-ImageJ (version 1.48d) software. For all constructs, we analysed cells with low to intermediate expression levels of talin. Expression levels were determined by fluorescence intensities measured across a large number of cells/FAs in cells exposed to the same amounts of fluorescent light (exposure time below 500 ms). Adhesion size measurements were performed using the particle analysis function of imageJ after careful background subtraction^7^. For cell circularity measurements, a freehand region of interest was drawn and circularity was measured. Cells were classified as being either ‘circular' or ‘elongated' as has been described previously, where elongated cells have a circularity ⩾0.56 (ref. 10). For ratio imaging experiments, all images were acquired using the same exposure times. Ratio images were created using custom software; briefly, background noise was removed by thresholding, followed by division of the vinculin immunofluorescence image by the GFP-talin image. For ratio quantification, GFP-talin and vinculin immunofluorescence images were background subtracted, a region of interest was drawn around individual, peripheral adhesions (7 per cell) and the integrated density was measured for both channels. A ratio value was produced for each adhesion by dividing the values from vinculin images by talin images.

### Fluorescence loss after photoactivation (FLAP)

Cells co-expressing the PAGFP-tagged protein of interest and the appropriate mCherry-tagged marker were plated onto fibronectin-coated glass bottom dishes. Both plasmids where expressed in a 1:1 ratio (cells were transfected with 1 μg of each) and only cells of healthy appearance with low to intermediate levels of the mCherry plasmid were selected for FLAP experiments. One hour before imaging, DMEM was replaced with Ham's F-12 medium supplemented with 25 mM HEPES buffer, 2% FCS, 1% penicillin/streptomycin and 1% L-glutamine. Cells were allowed to equilibrate in a pre-warmed environment at 37 °C with 5% CO_2_. Experiments were performed on a spinning disk confocal microscope (CSU10, Tokogawa) supplied by Intelligent Imaging Innovations, Inc. (3i), equipped with a 60 × /1.42 Plan Apo oil immersion objective (Zeiss). To photoactivate, a 405-nm laser was pulsed at full power for 5 ms at user-defined regions of interest. Fluorescence images were captured in 10 s intervals for a total period of 5 min using 488-nm and 561-nm laser lines used at full power with an appropriate exposure time. Approximately 4–5 FAs within a similar region of the cell were selected. To obtain intensity values over time, photoactivated fluorescence (PAF) images in the 488-nm channel were processed using a custom MATLAB (The Mathworks) script. Briefly, a user-defined threshold is used to detect FAs and create a mask. The mean intensity within each mask is measured and recorded. A second user-defined region is used to obtain background values, which are subtracted from the adhesion intensity values. Background-subtracted mean intensity values are then normalized to the first post-bleach intensities. Normalized intensity values were then fitted using Prism 6 (GraphPad Software) to single-phase exponential decay curves. The equation coefficients were extracted and transformed to generate F_M_ and t_1/2_ loss of PAF.

### Fluorescence recovery after photobleaching (FRAP)

Cells were transfected with GFP-tagged FA proteins of interest and FRAP experiments were performed using a Delta Vision RT microscope equipped with a 100 × /1.40 UPlan Apo oil immersion objective (Zeiss). FRAP laser beam diameter size was 1 μm and was held for 0.075 s per region of interest to bleach. Images were recorded every 10 s for 5 min following previously published methods^58^. Normalized fluorescence intensities were obtained using Softworx FRAP analysis software. Data were imported into MATLAB and fitted with single exponential curve fits according to published FRAP models^59^. A custom MATLAB script was used to extract the coefficients of the curve fits and the half-time (*t*_1/2_) of recovery and mobile fraction (MF) were calculated.

### Traction force microscopy

Fibronectin (FN) coated polyacrylamide (PAA) gels (6% of gel diluted from 30% Protogel in PBS, 37.5:1 fixed ratio of arylamide/bis-acrylamide; EC-890, National Diagnostics) containing 1:100 dilution of 0.2 μm FluoSpheres carboxylate-modified microspheres (F8805, blue fluorescent (365/415), Life Technologies) were prepared using previously published methods with modifications^60^,^61^. The stiffness (8.760±0.209 kPa) of 6% PAA gels were measured with a CellHesion Atomic Force Microscope (Nanowizard, CellHesion 200; JPK Instruments, Berlin, Germany) with tip-less cantilevers (NP-O10, Bruker AFM Probes) modified by attaching 10 μm diameter polystyrene beads (PPS-10.0, Kisker).

Transfected cells were plated on gels and allowed to spread overnight. Cell spreading deformed the surfaces of the gels thus changing the position of the embedded beads. Fluorescence images of the embedded beads were captured on a Delta Vision microscope system (Applied Precision, Washington, USA) using an oil immersion × 60 (NA=1.42) objective. After taking bead images, 0.05% Triton X100 was added to detach the cells and thus release forces mediated by cells on the gel. Subsequently, images of bead positions under null cellular forces were obtained as reference images. The traction forces exerted by cells were analysed by measuring the bead displacement induced by cells; i.e. bead position changes of reference image and image with cells. Traction force analysis was performed using a MATLAB algorithm^62^. Bead displacement was calculated using cross-correlation between both bead images (before and after Triton X100 treatment). The corresponding gel deformation was obtained by two-dimensional Gaussian interpolation. The stress field (Pa) was calculated from the gel deformation by implementing a Fourier transform-based algorithm using the Boussinesq Green's function. The total force (nN) was the sum of all overall traction forces exerted from one single cell.

### Protein expression and purification

Talin constructs were synthesized by PCR using a mouse talin1 cDNA as template, and cloned into pET-151/D-TOPO (Invitrogen). Constructs were expressed in *Escherichia coli* BL21 Star (DE3) cultured in LB. Recombinant His-tagged talin polypeptides were purified by nickel-affinity chromatography following standard procedures^63^. The His-tag was removed by cleavage with AcTEV protease (Invitrogen), and the protein was further purified by anion-exchange chromatography. Concentration was determined using extinction coefficients at 280 nm. Recombinant His- tagged chicken vinculin domain 1 (Vd1; residues 1–258) was expressed from a pET-15b plasmid and purified via nickel-affinity chromatography followed by anion-exchange chromatography, as described by^20^.

### Actin co-sedimentation assays

Rabbit skeletal muscle G-actin^64^ was polymerized in 10 mM Tris, 50 mM NaCl, 100 μM ATP, 1 mM DTT, 1 mM MgCl2, pH 7.0. Assays were performed using 4 μM talin polypeptides and 10 μM F-actin. The mixture was incubated for 60 min at room temperature and centrifuged at 100,000 r.p.m. for 30 min at 22 °C using a Beckman Optima TM ultracentrifuge. Supernatants and pellets were analysed on 12% SDS–PAGE gels and stained using Coomassie blue.

### FACS Analysis of integrin activation

The level of β1 integrin activation was measured by staining cells with the 9EG7 monoclonal antibody (553715, BD Biosciences) and fluorescence activated cell sorting (FACS). Cells (10^6^/tube) were washed and re-suspended in FACS buffer (1% bovine serum albumin (BSA: A9647, Sigma) in PBS), incubated with 9EG7 diluted in FACS buffer for 1 h on ice and then washed in the same buffer three times. Cells were then incubated with DyLight 649 conjugated donkey anti-rat (Jackson lmmunoResearch) diluted in FACS buffer for 45 min on ice before two washes with FACS buffer, and a final wash with PBS. FACS analysis was carried out by using Cyan ADP (Beckman Coulter) and Summit software (V4.3, Beckman).

## Additional information

**How to cite this article:** Atherton, P. *et al*. Vinculin controls talin engagement with the actomyosin machinery. *Nat. Commun.* 6:10038 doi: 10.1038/ncomms10038 (2015).

## Supplementary Material

Supplementary Figures Supplementary Figures 1-9

Supplementary Movie 1 Related to Figure 1. Recording of TKO spreading on fibronectin-coated glass. The video shows a TKO cell transfected with GFP-talinFL spreading on fibronectin-coated glass over two hours. Several non-transfected cells can be seen within the same field-of-view, which do not spread and remain rounded. Imaging was started prior to the addition of cells to fibronectin-coated glass in a microscope chamber kept at 37 °C supplemented with 5% CO_2_. Images were acquired every 150 seconds for 2 hours. Images are played back at 5 frames/s. Scale bar represents 40 μm.

Supplementary Movie 2 Related to Figure 2. Recording of NIH3T3 cells to demonstrate the fluorescence loss after photoactivation (FLAP) technique. The video shows an NIH3T3 cell expressing zyxin-mCherry and photoactivatable (PA)GFP-talinFL before and after photoactivation with a 405-nm laser. The box indicates the region that was targeted for photoactivation and the fluorescence intensity plot shows the FLAP over time. Images were taken every 10 seconds for 5 minutes following photoactivation. Images are played back at 5 frames/s. Scale bar represents 10 μm.

Supplementary Movie 3 Related to Figure 2. Recording of FLAP in FAs from NIH3T3 cells expressing the indicated PAGFP-talin construct +/- constitutively active vinculin. The top panel shows FLAP in control NIH3T3 cells. The fluorescence intensity plot shows the FLAP over time. Note the increased turnover of talΔR4-R10 and talΔR1-R10 compared to talinFL. The bottom panel shows FLAP in NIH3T3 cells co-expressing vinT12. Note the reduced turnover of talinFL and talΔR4-R10, but not talΔR1-R10, in the presence of constitutively active vinculin. Images were taken every 10 seconds for 5 minutes following photoactivation. Images are played back at 5 frames/s. Scale bar represents 5 μm.

Supplementary Movie 4 Related to Figure 2. Recording of FLAP in FAs from NIH3T3 cells expressing PAGFP-talinFL +/- vinT12 or vinT12^A50I^. The video shows FLAP in NIH3T3 cells expressing PAGFP-tagged talinFL as well as the indicated constitutively active mCherry-tagged vinT12 construct. Note that vinT12, but not vinT12^A50I^, is able to stabilise talinFL within FAs. Images were taken every 10 seconds for 5 minutes following photoactivation. Images are played back at 5 frames/s. Scale bar represents 5 μm.

Supplementary Movie 5 Related to Figure 3. Recording of FLAP in FAs from TKO cells expressing PAGFP-talinFL or talΔR2R3. The video shows FLAP in TKO cells expressing PAGFP-tagged talinFL or talΔR2R3. The fluorescence intensity plot shows the FLAP over time. Note the reduced turnover of talΔR2R3 compared to talinFL. Images were taken every 10 seconds for 5 minutes following photoactivation. Images are played back at 5 frames/s. Scale bar represents 5 μm.

Supplementary Movie 6 Related to Figure 5. Recording of FLAP in FAs from TKO cells expressing PAGFP-talinFL or talΔR8. The video shows FLAP in TKO cells expressing PAGFP-tagged talinFL or talΔR8. The fluorescence intensity plot shows the FLAP over time. Note the increased turnover of talΔR8 compared to talinFL. Images were taken every 10 seconds for 5 minutes following photoactivation. Images are played back at 5 frames/s. Scale bar represents 5 μm.

Supplementary Movie 7 Related to Figure 5. Recording of FLAP in FAs from TKO cells expressing PAGFP-talinFL or talinFL^ABS2mut^ +/- constitutively active vinculin. The top panel shows FLAP in control TKO cells. The fluorescence intensity plot shows the FLAP over time. Note the increased turnover on talinFLABS2mut compared to talinFL. The bottom panel shows FLAP in TKO cells co-expressing vinT12. Both talinFL and talinFL^ABS2mut^ show a reduced turnover in the presence of constitutively active vinculin. However, talinFL^ABS2mut^ is still more mobile than talinFL. Images were taken every 10 seconds for 5 minutes following photoactivation. Images are played back at 5 frames/s. Scale bar represents 5 μm.

Supplementary Movie 8 Related to Figure 6. Recording of FLAP in FAs from TKO cells expressing PAGFP-talΔR2R3 or talΔR2R3^ABS2mut^. The video shows FLAP in TKO cells expressing PAGFP-tagged talΔR2R3 or talΔR2R3^ABS2mut^. The fluorescence intensity plot shows the FLAP over time. Note the increased turnover of talΔR2R3^ABS2mut^ compared to talΔR2R3. Images were taken every 10 seconds for 5 minutes following photoactivation. Images are played back at 5 frames/s. Scale bar represents 5 μm.

Supplementary Movie 9 Related to Figure 6. Recording of FLAP in FAs from TKO cells expressing PAGFP-talinFL +/- vinT12 or vinT12^I997A^. The video shows FLAP in TKO cells expressing PAGFP-tagged talinFL as well as the indicated constitutively active vinT12 construct. Note that in the presence of vinT12^I997A^, talinFL is partially stabilised within FAs, but not to the same level as with vinT12. Images were taken every 10 seconds for 5 minutes following photoactivation. Images are played back at 5 frames/s. Scale bar represents 5 μm.
