# Supplementary material for: Vinculin controls talin engagement with the actomyosin machinery
Source: Nat Commun. 2015 Dec 4;6:10038. doi: 10.1038/ncomms10038 (PMC4686655; doi:10.1038/ncomms10038)
Supplement: Supplementary Figures — 1-9 [file ncomms10038-s1.pdf]

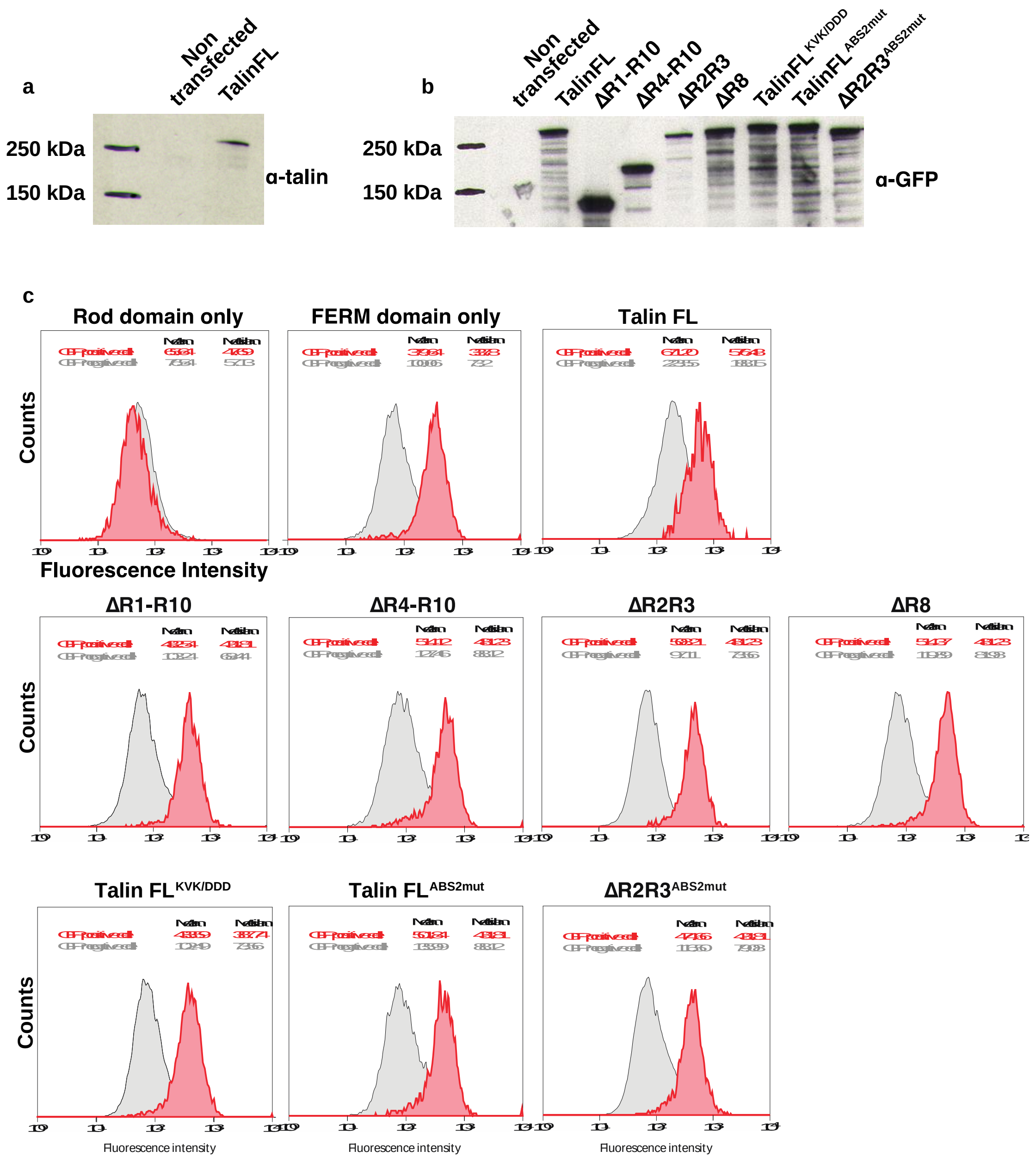

Supplementary Figure 1. A. Left panel: Western blot from lysates of non-transfected and GFP-talinFL expressing TKO cells; blot was probed with an anti-talin antibody (8d4) for talin detection. **B**. Western blot from lysates of NIH3T3 cells expressing indicated GFP-tagged talin constructs. Blot was probed with an α-GFP antibody for the detection of the different forms of talin. Highest intensity bands have the expected MW of the expressed talin polypeptides **C**. Integrin activation profiles measured using FACS analysis. Red profile is of cells expressing the indicated GFP-fusion constructs, grey is from untransfected cells in the same samples. Note that all the talin constructs containing the FERM domain activate integrins to a similar level while the talin rod only is unable to activate integrins.

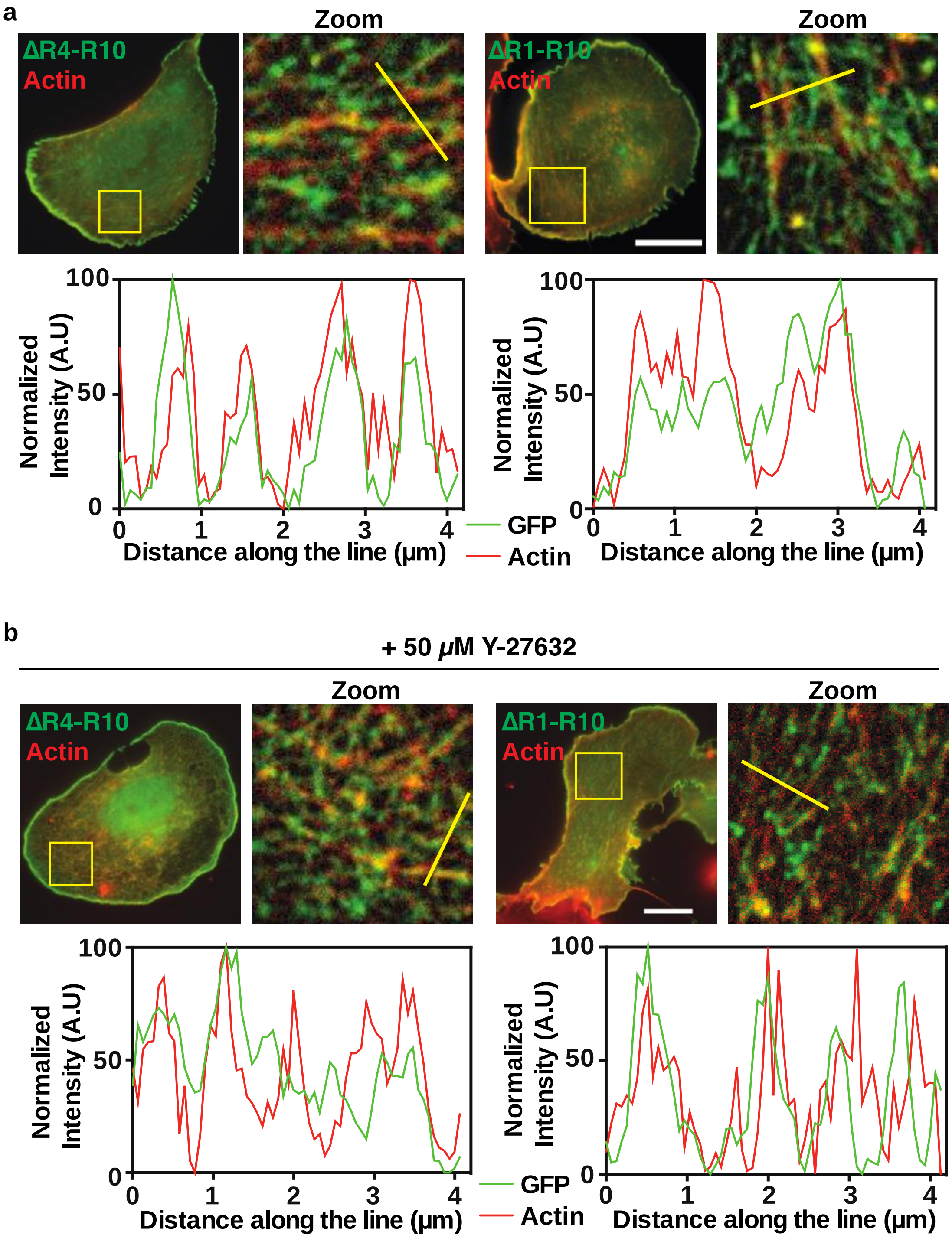

Supplementary Figure 2. A. Talin ΔR4-R10 (talΔR4-R10) and ΔR1-R10 (talΔR1-R10) constructs colocalize with thin actin filaments B**.** Colocalization of talΔR4-R10 and talΔR1-R10 with actin filaments is maintained after treatment with Y-27632. Scale bar represents 10 µm.

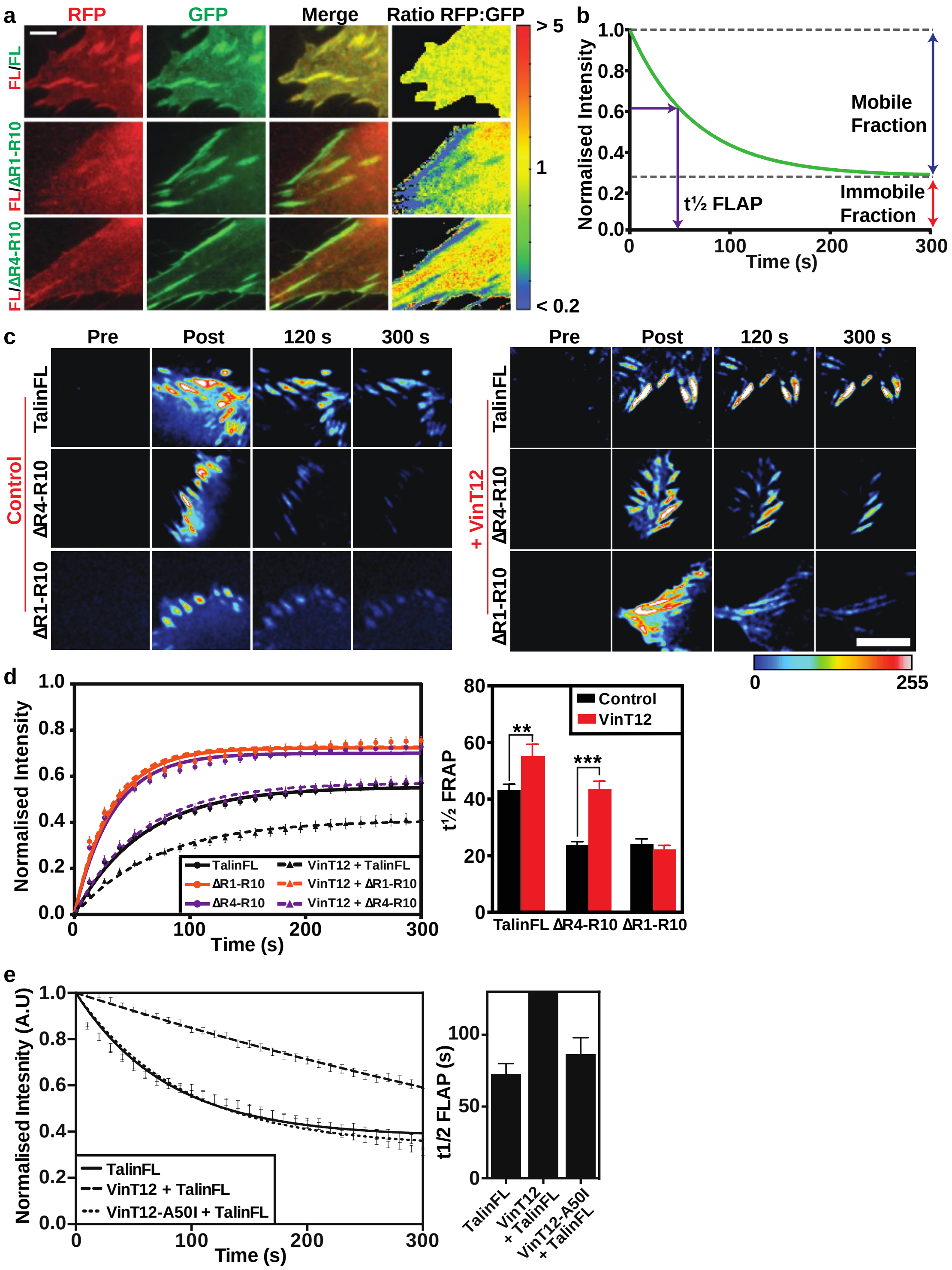
a
RFP
GFP
Merge
Ratio RFP:GFP
> 5
1
< 0.2
FL/FL
FL/ΔR1-R10
FL/ΔR4-R10
b
Normalised Intensity
Mobile Fraction
Immobile Fraction
t½ FLAP
Time (s)
c
Pre
Post
120 s
300 s
TalinFL
ΔR4-R10
ΔR1-R10
Control
+ VinT12
0
255
d
Normalised Intensity
Time (s)
TalinFL
ΔR1-R10
ΔR4-R10
VinT12 + TalinFL
VinT12 + ΔR1-R10
VinT12 + ΔR4-R10
t½ FRAP
Control
VinT12
**
***
TalinFL
ΔR4-R10
ΔR1-R10
e
Normalised Intesnity (A.U)
Time (s)
TalinFL
VinT12 + TalinFL
VinT12-A50I + TalinFL
t1/2 FLAP (s)
VinT12 + TalinFL
VinT12-A50I + TalinFL

Supplementary Figure 3. A. Representative images of NIH3T3 cells co-expressing GFP-tagged talin-FL and talin deletion constructs with RFP-talinFL. Note that the expression of talin deletion mutants leads to a decreased presence of talinFL in FAs, suggesting that the mutants partially outcompete talinFL for binding in FAs. Scale bar represents 5 µm. **B.** Scheme of a Fluorescence Loss After Photoactivation (FLAP) curve. **C.** Images taken from FLAP movies colour coded for fluorescence intensity (high intensity white, low intensity blue). Loss of fluorescence of talinFL from FAs in NIH3T3s is slower compared to talΔR4-R10 and talΔR1-R10. Co-expression of vinT12 retards loss of fluorescence for talinFL and talΔR4-R10 but not talΔR1-R10. Scale bar represents 5 µm. **D.** The same experiment as in C was repeated using Fluorescence Recovery After Photobleaching (FRAP) showing a similar pattern to that seen when using FLAP (error bars are +/- SEM, n < 60 FAs, ** = $p < 0.01$, *** = $p < 0.001$ (ANOVA)). **E.** Fluorescence loss after Photoactivation (FLAP) experiments in NIH3T3 cells expressing talinFL and talinFL in the presence of vinT12 or vinT12^{A50I}. Note that the vinT12^{A50I} talin binding mutant is unable to stabilise talinFL in FAs.

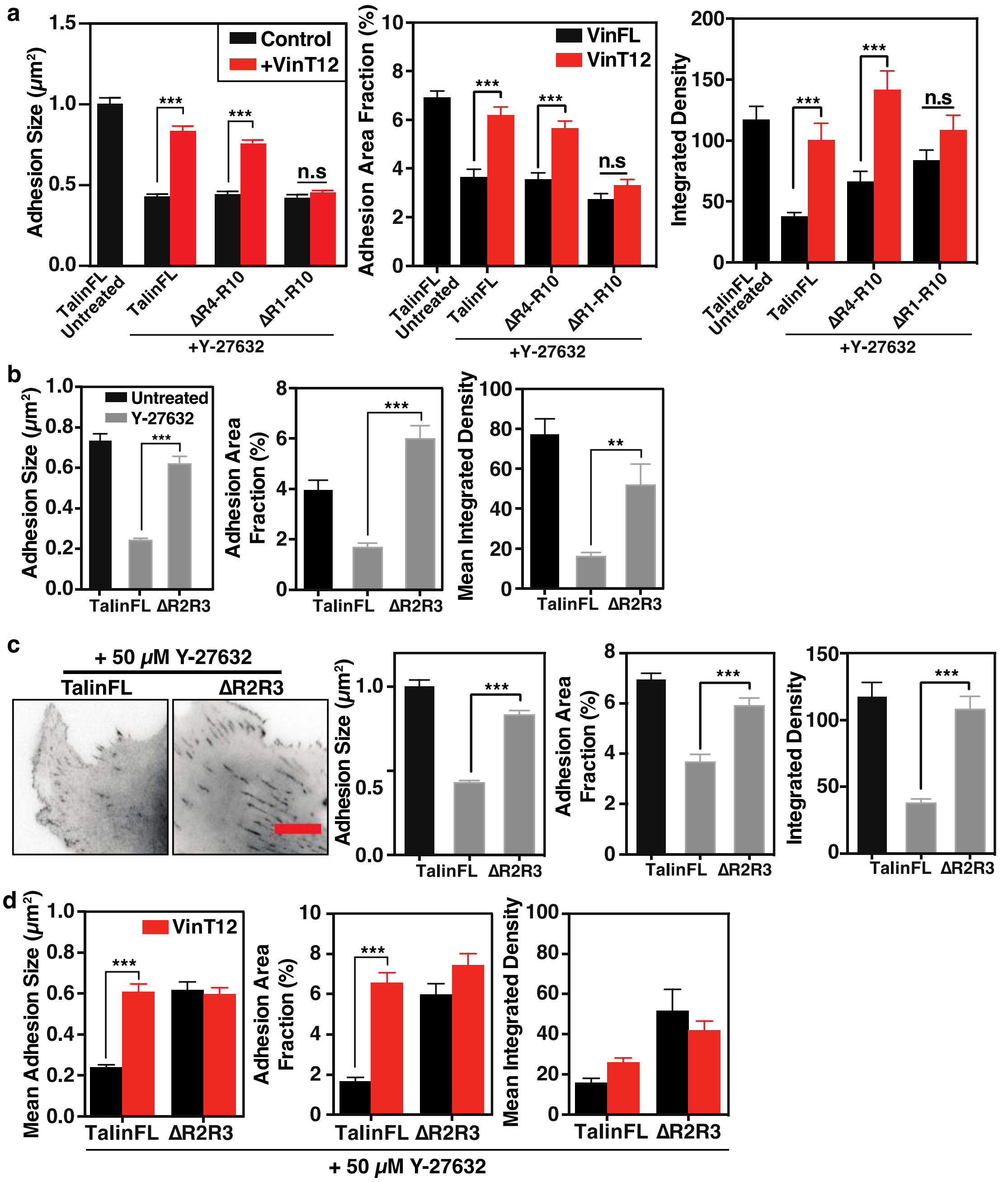

Supplementary Figure 4. Quantification of adhesion size, adhesion area fraction and integrated density of adhesions in **A.** NIH3T3 cells co-expressing either vinFL or vinT12 with the indicated talin construct after Y-27632 treatment (error bars are +/- SEM, n < 60 FAs, ** = p < 0.01. *** = p < 0.001 (ANOVA)); **B.** TKO cells expressing either talinFL or talΔR2R3 after Y-27632 treatment; **C.** NIH3T3 cells co-expressing either talinFL or talΔR2R3 with vinFL after Y-27632 treatment (scale bar represents 10 µm); **D.** TKO cells expressing either talinFL or talΔR2R3 with/without co-expression of vinT12 after Y-27632 treatment. Error bars are +/- SEM, n < 60 FAs, ** = p < 0.01. *** = p < 0.001 (t-test).

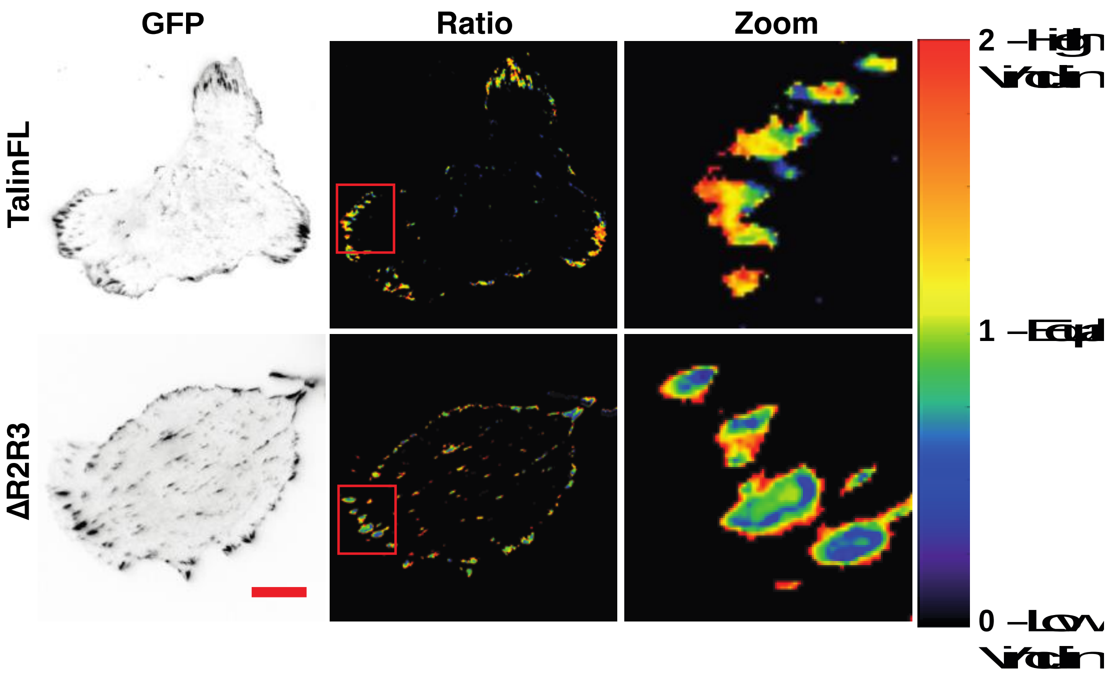

Supplementary Figure 5. Images of vinculin:talin ratios in FAs of TKO cells expressing either TalinFL or talΔR2R3 that were stained with an antibody for the detection of endogenous vinculin. Note that FAs of cells expressing talΔR2R3 have reduced vinculin:talin ratio compared to cells expressing talinFL. Scale bar represents 10 µm.

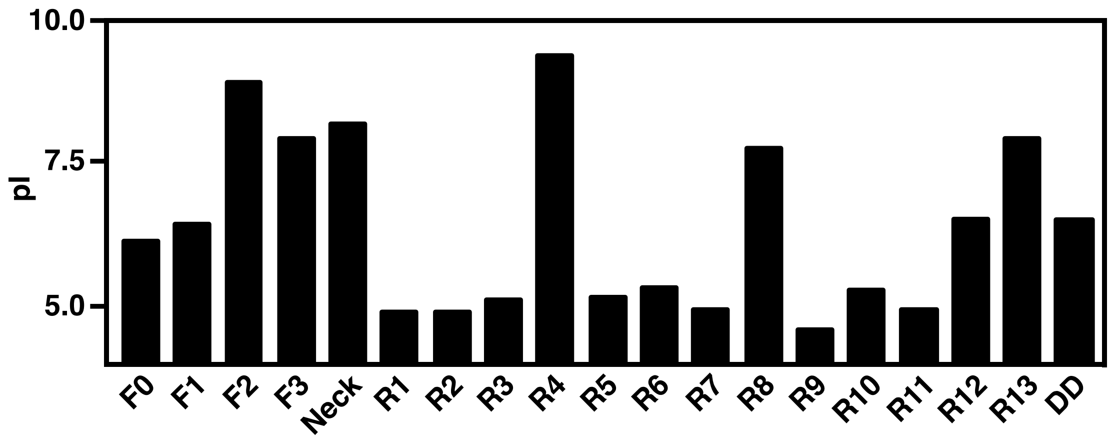

Supplementary Figure 6. Theoretical isoelectric point (pI) of each of the talin domains calculated using ProtParam (http://web.expasy.org/protparam/protpar-ref.html).

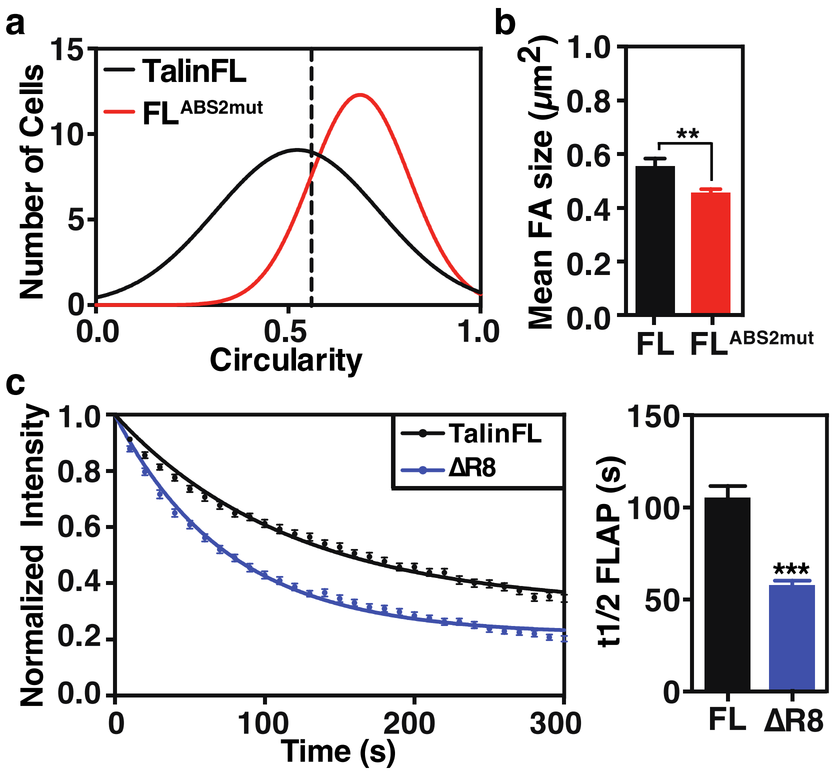

Supplementary Figure 7. A. Circularity histograms for talinFL and talinFL^{ABS2mut} expressed in TKO cells. Expression of talinFL^{ABS2mut} results in more circular cells. **B.** FAs of TKO cells expressing talinFL^{ABS2mut} are smaller than those of cells expressing talinFL (error bars are +/- SEM, n = 60 cells, ** = $p < 0.01$ (t-test)). **C.** FLAP experiments in TKO cells expressing either talinFL or talΔR8 show that the talΔR8 construct has a faster turnover compared to talinFL (error bars are +/- SEM, n = 30 – 60 FAs from 14 - 19 cells, ** = $p < 0.01$ (t-test)).

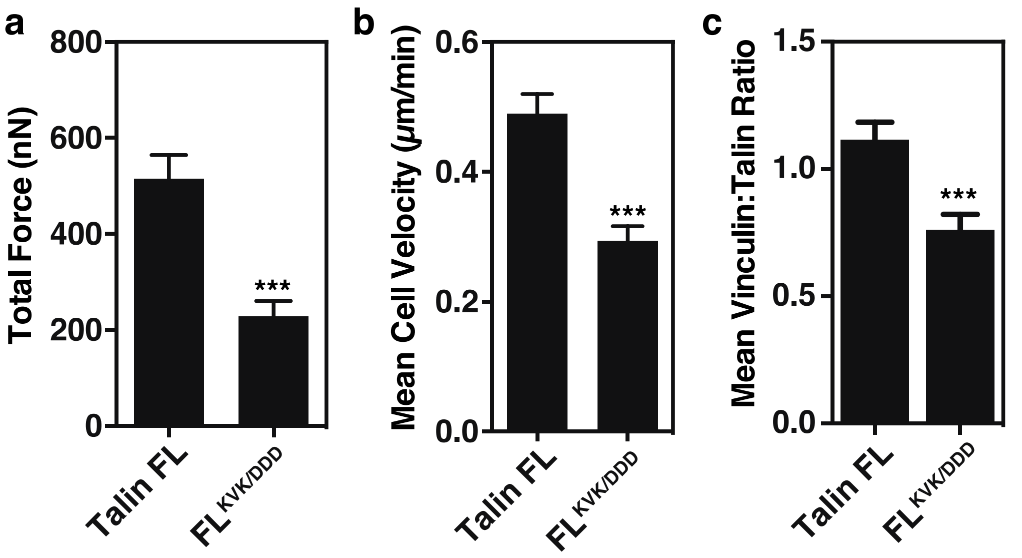

Supplementary Figure 8. A. Measurements of total force exerted by TKO cells expressing either talinFL or the talinFL$^{KVK/DDD}$ ABS3 mutant. Cells expressing talinFL$^{KVK/DDD}$ exert less force than those expressing talinFL (error bars are +/- SEM, n = 33 (talinFL) and 35 (talinFL$^{KVK/DDD}$) cells, *** = $p < 0.01$ (t-test)). **B**. TKO cells expressing talinFL$^{KVK/DDD}$ migrate slower than those expressing talinFL (error bars are +/- SEM, n = 23 (talinFL) and 32 (talinFL$^{KVK/DDD}$) cells, *** = $p < 0.01$ (t-test)). **C**. FAs of talin-/- cells expressing talinFL$^{KVK/DDD}$ have reduced vinculin present compared to those of cells expressing talinFL (error bars are +/- SEM, n = 60 FAs from 20 cells, *** = $p < 0.01$ (t-test)).

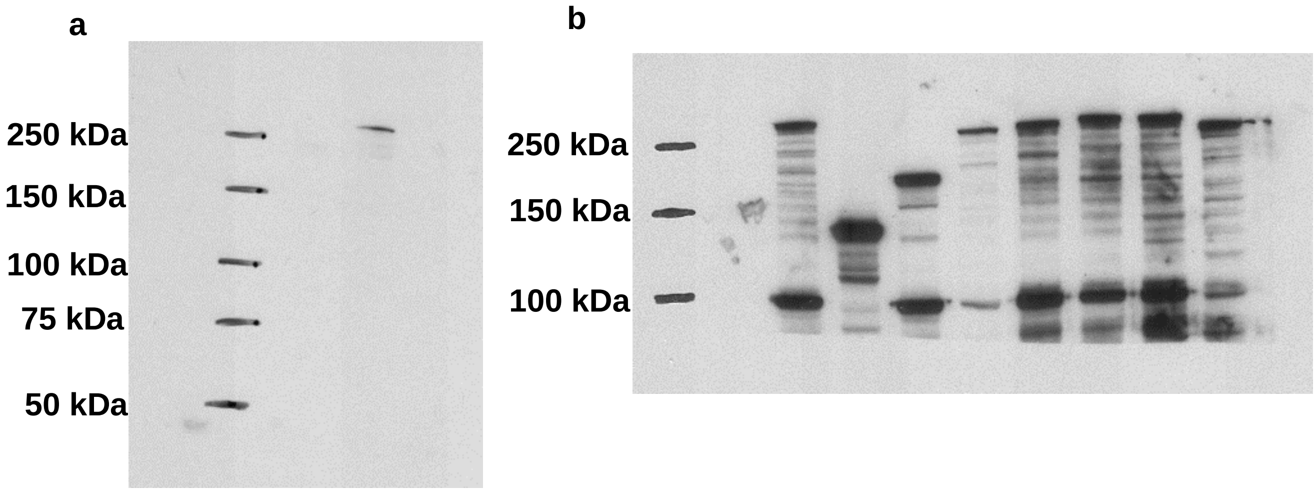

Supplementary Figure 9. A. Uncropped western blot originally shown in Supplementary Figure 1A. **B.** Uncropped western blot originally shown in Supplementary Figure 1B.
